# Extensive simulations assess the performance of genome-wide association mapping in various *Saccharomyces cerevisiae* subpopulations

**DOI:** 10.1098/rstb.2020.0514

**Published:** 2022-07-18

**Authors:** Jackson Peter, Anne Friedrich, Gianni Liti, Joseph Schacherer

**Affiliations:** ^1^ Université de Strasbourg, CNRS, GMGM UMR 7156, Strasbourg, France; ^2^ Université Côte d'Azur, CNRS, INSERM, IRCAN, Nice, France; ^3^ Institut Universitaire de France (IUF), Paris, France

**Keywords:** genome-wide association study, population genomics, complex trait, variant mapping, yeast

## Abstract

With the advent of high throughput sequencing technologies, genome-wide association studies (GWAS) have become a powerful paradigm for dissecting the genetic origins of the observed phenotypic variation. We recently completely sequenced the genome of 1011 *Saccharomyces cerevisiae* isolates, laying a strong foundation for GWAS. To assess the feasibility and the limits of this approach, we performed extensive simulations using five selected subpopulations as well as the total set of 1011 genomes. We measured the ability to detect the causal genetic variants involved in Mendelian and more complex traits using a linear mixed model approach. The results showed that population structure is well accounted for and is not the main problem when the sample size is high enough. While the genetic determinant of a Mendelian trait is easily mapped in all studied subpopulations, discrepancies are seen between datasets when performing GWAS on a complex trait in terms of detection, false positive and false negative rate. Finally, we performed GWAS on the different defined subpopulations using a real quantitative trait (resistance to copper sulfate) and showed the feasibility of this approach. The performance of each dataset depends simultaneously on several factors such as sample size, relatedness and population evolutionary history.

This article is part of the theme issue ‘Genetic basis of adaptation and speciation: from loci to causative mutations’.

## 1. Introduction

One key concern in biology is to understand what drives the phenotypic diversity observed between individuals, populations and species. In this context, being able to find genetic variants responsible for this diversity is an important step toward the elucidation of the genetic architecture of traits. Due to the emergence of cost-effective sequencing technologies, the generation of large-scale sequence datasets for a large number of individuals of the same species no longer represents a major bottleneck, in particular for model organisms. To this end, the last decade has seen the initiation of several large-scale resequencing projects, enabling the gathering of enough sequences to make high-throughput approaches possible. Large-scale polymorphism surveys have been reported in humans [1,2], *Arabidopsis thaliana* [3], *Saccharomyces cerevisiae* [4] and *Caenorhabditis elegans* [5], for example. Their principal goal was to gather a large number of genome sequences to establish a catalogue of genetic variants, including rare ones.

There are multiple motivations for carrying out large-scale population genomics projects. First, they are designed to enable a better understanding of the demographic and evolutionary histories of populations. Second, such datasets provide insight into the processes by which genetic diversity is generated and maintained. Third, they can help to distinguish effects acting on the entire genome (such as drift, migration, inbreeding) and those acting on individual loci (selection, mutation, recombination). Finally, one of the major incentives of resequencing projects is to gain a better understanding of the relationship between genotypes and phenotypes, and more specifically to build genetic datasets that would make it possible to map allelic variants responsible for phenotypic diversity.

One way to access the genotype-phenotype relationship is to conduct genome-wide association studies (GWAS). This strategy has become an elegant approach to dissect natural variation by associating phenotypes with genotypes at the genome scale as it has the advantage of benefitting from the historical recombination in wild populations to detect non-random associations between phenotype of interest and genetic variants distributed throughout the genome. To date, many studies have already proven that GWAS is a very effective way to find genetic determinants associated with quantitative traits. These studies have been conducted primarily on model organisms, and investigated traits mostly focus on human genetic diseases [6,7], agronomy or industrially relevant traits like resistance to chemical compounds, production of oil for *Arabidopsis thaliana* [8–10], grain yield under water deficit on *Oryza sativa* [11], or longevity using the DGRP dataset for *Drosophila melanogaster* [12]. The power of these kind of studies comes with the integration of multi-omics data [13]. However, this strategy has some major limitations including the important multiple testing burden, the modest proportion of the estimated heritability explained by associated genetic variants, and the difficulty in detecting common variants with small effects or rare causal variants.

Despite being a widely used model organism for genetics, yeasts, and more precisely *S. cerevisiae,* are under-represented model organisms for GWAS. This is mainly due to the fact that a large number of natural isolates have only recently been sequenced [4]. They are nonetheless ideal to conduct surveys of population genomics for several reasons. First, their genomes are small and compact (around 12 Mb) and are therefore relatively easy to sequence. Second, yeast can be isolated from a broad range of ecological and geographical origins, thus maximizing the genetic and phenotypic diversity within the studied species. Finally, regarding the dissection of the genotype–phenotype relationship, yeasts have the advantage of forming clonal colonies, allowing phenotypic measurements to be replicated, enabling multiple surveys and generating reproducible data. Until a few years ago, only a hundred genome sequences were available, limiting the possibility of conducting GWAS. However, several studies aimed to evaluate the feasibility of genome-wide association on *S. cerevisiae* and try to appreciate its limits [14–16]. Some limitations were assessed, such as the inflated type-I error (i.e. false positive) due to the population stratification and the resulting challenges in separating signal from noise, which highlighted the importance of efficiently correcting this confounding factor when performing GWAS. Although these studies laid the groundwork for further associations within *S. cerevisiae*, they all suffered from the lack of statistical power due to the limited size of the cohort used. The genomic sequences of 100 strains associated with 49 phenotypes were used to perform GWAS and made it possible to find associations representing proof of principle, with a high probability of detecting large-effect loci, but emphasizing that 100 genomic sequences still limit the power of analyses, with little or no significant association found for multiple phenotypes [17,18].

More recently, sequencing efforts have been carried out and allowed for the availability of more than 2000 *S. cerevisiae* genome sequences [4,17,19,20]. Of the different datasets, the largest [4] aimed to obtain deep coverage sequenced genomes for more than 1000 natural isolates of *S. cerevisiae*. This survey highlighted differential genome evolution patterns across 26 subpopulations with their own and unique evolutionary history. In addition, the dataset was large enough to overcome aforementioned statistical power issues and was found to be suitable for performing GWAS, as genetic variants were significantly associated with 14 traits, most of them being complex (i.e. involving more than one locus) [4]. To get a better idea of the variability of the performance across subpopulations, we decided to test whether certain subpopulations or subsets of genomes will reduce certain biases in the parameters that influence the outcome of such associations. Here, we simulated phenotypes based on different genetic architectures, i.e. governed by one single nucleotide polymorphism (SNP) for a Mendelian trait and by 10 SNPs for a complex trait. We then measured our ability to identify the causal variants via GWAS on five subsets of the 1011 genomes as well as the full dataset (see Material and methods). Our results suggest that associations are easily found for Mendelian traits, with the exception of one dataset showing a very high type-I error rate due to cryptic relatedness. Regarding the association with more complex phenotypes, performance varies considerably between the cohorts used, with some causal variants left unidentified due to their small effect size. Together, these results underscore the need for careful selection of individuals from the total dataset when running GWAS. The size of the population is of great importance, as predicted by previous studies, but the presence of confounding factors sometimes leads to unreliable results. Certain phenotypic properties also modify the power of detection, such as the effect size or the complexity of the phenotype. Finally, we present an example of an association using the copper sulfate resistance phenotype, a well-known simple trait in *S. cerevisiae*, to see if the results follow our simulations.

## 2. Material and methods

### (a) *Saccharomyces cerevisiae* isolates, sequencing data and reads mapping

All the strains and sequencing data used for this study were obtained from Peter *et al.* [4]. The individual cleaned VCF (variant call format) files constructed in the context of this project were directly used for further analyses.

### (b) Subpopulations and datasets tested

For this study, we worked with six different datasets, based on 1011 previously sequenced isolates of *S. cerevisiae* [4]:

— the complete dataset, that gathers the 1011 isolates,
— a dataset named ‘sampled diversity’ that is composed of 133 isolates representative of the genetic diversity of the *S. cerevisiae* species and for which the strains were selected to avoid overrepresentation of some specific groups (electronic supplementary material, table S1),
— four datasets selected according to the subpopulations defined in Peter *et al.* [4] and composed of nearly 50 or more isolates: the European wine dataset that gathers 323 strains related to winemaking, the sake dataset with 47 isolates used for sake fermentation, the mosaic region 3 that groups 113 isolates and the mixed origin cluster, which brings together 71 wild and industrial—mostly bakery—strains.

### (c) Genome-wide association studies matrix preparation

The joint calling method of GATK [21] was run with the individual cleaned VCF files to create a complete genotyping matrix for each of the six datasets used in this study.

For each of these, we used vcftools [22] to keep only the biallelic SNPs, by setting the min-alleles and the max-alleles to 2. The average pairwise nucleotide diversity π was estimated based on this matrix with the --window-pi option, considering the whole genome as window size.

We also used vcftools to filter missing genotypes as follows with an arbitrary threshold to exclude all variants present in less than 1000 individuals for the total matrix (--max-missing-count option has been set to 11), and we excluded all sites with missing calls for the subset matrices (--max-missing 1.0 to allow only sites with 100% present calls). Finally, we excluded from the matrices the sites that had a Minor Allele Frequency (MAF) < 5%, using PLINK 1.9 with the --maf option set to 0.05 [23].

The size of the constructed matrices is recapitulated in table 1.

**Table 1.** Datasets description.

datasets	individuals	*π*	polymorphic positions	singletons	biallelic polymorphic positions, no missing	biallelic polymorphic positions, no missing, MAF > 5%
1011 strains	1011	0.0044	1 625 809	509 011	1 346 007	82 869
mixed origins	71	0.0032	142 093	3959	97 690	81 030
mosaic region 3	113	0.0042	496 841	174 079	365 433	72 807
sake	47	0.0008	100 257	14 548	84 197	21 489
sampled diversity	133	0.0049	935 060	506 761	720 709	66 299
European wine	323	0.001	284 342	105 123	218 789	14 164

Copy number variants (CNVs) detected in Peter *et al.* [4] were added in the complete matrix. We first converted CNV information using the plink recode12 option, which allowed us to encode the CNVs with 1 corresponding to the presence of the gene with one copy only, and 2 indicating that the gene is amplified. This encoding allowed us to add 925 CNVs that showed variation among the 1011 isolates.

### (d) Phenotype simulation

For each of the six datasets, we simulated 1000 Mendelian traits (governed by a single causal SNP) and 1000 complex traits (governed by 10 SNPs). Causal SNPs were randomly chosen in the SNP matrix and a phenotype was generated accordingly using GCTA [24]. The heritability of all the simulated traits was chosen to be 0.8 for each dataset. The command executed for the phenotype simulations was the following:

gcta --bfile $snp --maf $maf --simu-qt --simu-causal-loci $snplist --simu-hsq 0.8 --simu-rep 1 -out $output

With the simulation of a complex trait, we used GCTA's default effect size assignation method, which comprises generating them from a standard normal distribution among the 10 causal SNPs.

### (e) Phenotyping on CuSO_4_

Quantitative high-throughput phenotyping was performed using endpoint colony growth on solid media [25]. Strains were pregrown in flat bottom 96-well microplates containing liquid YPD medium. The replicating ROTOR HDA© benchtop robot (Singer instruments) was used to mix and pin strains onto a solid YPD matrix plate at a density of 384 wells. The matrix plates were incubated overnight at 30°C to allow sufficient growth and replicated on CuSO_4_ 10 mM as well as on YPD 30°C as pinning and growth control. Each isolate was present in quadruplicates on the corresponding matrix (interplate replicates) and at two different positions (intraplate replicates). The plates were incubated at 30°C for 40 h and were scanned at a resolution of 600 dpi and 16-bit grayscale. Quantification of the colony size from plate images was performed using the software package Gitter [26]. Each value was normalized using growth ratio between the stress media and standard YPD medium 30°C.

### (f) Estimation of the genome-wide heritability

The estimation of genome-wide heritability was completed by dividing the genetic variance of the null model by the total variance of the null model (genetic variance and residual variance), computed using FaST-LMM.

### (g) Association

We performed mixed-model association analysis using FaST-LMM [27]. The command used for association was the following:

fastlmmc -bfile $snp -bfileSim $snp -pheno $pheno -out $assoc_file -verboseOutput

The mixed model adds a polygenic term to the standard linear regression designed to circumvent the effects of relatedness and population stratification. To quantify the extent of the bulk inflation and the excess false positive rate, we computed the genomic inflation factor *λ* for each run of simulation. This factor is defined as the ratio between the median of the empirically observed distribution of the test statistic on the expected median. For example, the *λ* for a standard allelic test for association is based on the median (0.456) of the 1 d.f. *χ*^2^ distribution. Under a null model of no association and unlinked variants, the expectation is for the *λ* to be 1. A *λ* superior to 1 indicates inflated *p*-values of association, possibly due to a confounding factor not accounted for.

### (h) Permutations and simulations evaluation

We estimated a trait-specific *p*-value threshold for each condition by permuting phenotypic values between individuals 100 times. The significance threshold was the 5% quantile (the fifth lowest *p*-value from the permutations). With that method, variants passing this threshold have a 5% family-wise error rate. In order to evaluate and compare the power of our datasets to recover causal SNP(s), we built a table of confusion for each run of simulation, by measuring the number of true positives (TP), true negatives (TN), false positives (FP) and false negatives (FN), transposed into rates with the following formulas:

— True positive rate (TPR) = TP/(TP + FN)
— True negative rate (TNR) = TN/(TN + FP)
— False positive rate (FPR) = FP/(FP + TN)
— False negative rate (FNR) = FN/(FN + TP)

## 3. Results

### (a) Characteristics of subpopulations selected for genome-wide associations

**Figure 1.**
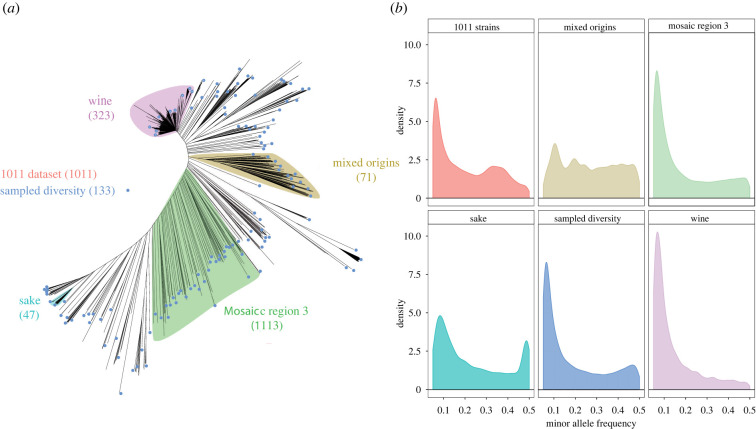
Overview of the six datasets used in this study. (*a*) Phylogenetic relationships between the 1011 *S. cerevisiae* isolates, illustrated by a neighbour-joining tree constructed with all biallelic SNPs in the population [4]. Branches of the four phylogenetic clusters are highlighted with different colours while the isolates from the sampled diversity dataset are designed with a blue circle. (*b*) Distribution of the minor allele frequency of the polymorphic positions within the six considered datasets.

The performance of genome-wide association studies varies greatly depending on the characteristics of the population used to conduct the experiment. In order to evaluate the influence of parameters such as minor allele frequency, sample size and relatedness, we used six different sets of individuals from the 1011 sequenced isolates of *S. cerevisiae*. Among the different clades identified though the analysis of phylogenetic relationships between the isolates [4], four were directly selected as subsets: European wine, sake, mosaic region 3 and the mixed origin cluster (figure 1*a*). We have also included the complete dataset with 1011 individuals and the sampled diversity dataset composed of 133 individuals representing the genetic diversity of the *S. cerevisiae* species. These datasets vary in size, both in terms of number of individuals and polymorphic sites (table 1), but their performance is probably not solely related to these characteristics, as multiple factors are expected to influence the results of the associations. As for other species such as humans or *A. thaliana*, there is a bias toward low-frequency variants in *S. cerevisiae*, which can be illustrated by the 31.3% of singletons, i.e. variants that are only found in one isolate, as observed in the 1011 strains [4]. Across the different defined subsets, this value is highly variable, ranging from 2.8% for the mixed origins cluster to 54.2% for the sampled diversity dataset, while the European wine and mosaic region 3 datasets have a proportion of singletons close to the entire population (table 1).

In order to avoid false associations, successive quality control filtering steps were applied to the SNP matrices generated for each dataset. We kept only biallelic SNPs and filtered out SNPs with missing genotypes for all matrices, except for the 1011 strains dataset, for which we kept SNPs with at least 1000 informed genotypes. The markers with a MAF lower than 5% were also removed. The retained matrices contain between 14 164 SNPs for the European wine group and 82 869 SNPs for the 1011 strains and cover the entire genome in each dataset (table 1). After these pruning steps, the distribution of the MAF shows a rapid decrease, with a strong bias toward the low frequency variants (figure 1*b*). However, the distribution of the MAF is more uniform for the mixed origins dataset, and the sake dataset has 13% of the SNPs with a MAF of around 0.49. These latter SNPs are distributed across the entire genome and this bias is linked to the fact that half of the sake strains are clustered on a thin branch of the tree, suggesting that they are very close and share a recent common ancestor (figure 1*a*).

In order to test the ability of each dataset to map the causal variants responsible for phenotypic variation, extensive simulations were performed. The causal genetic determinants of two types of traits, Mendelian and complex traits governed by one and a large number of genetic variants of varying effect sizes respectively, were selected at random. These simulated traits obviously do not summarize all the complexity of the genetic architecture of the traits but give an overview of the behaviour of the different populations on the GWAS performance according to the type of trait. Phenotypic data were generated based on the genotype of each strain. The association was run using FaST-LMM, which adds a polygenic term to the standard linear regression designed to circumvent the effects of kinship and population stratification [27]. These steps were repeated 1000 times for each type of trait and dataset. For each run, the phenotype dataset was permuted 100 times and the lowest *p*-value of each genome-wide test was recorded. And a genome-wide specific significance threshold of 5% family-wise error rate was estimated.

### (b) Detection and mapping of Mendelian traits in the different subpopulations

We first assessed the ability to detect an association between the different matrices and a Mendelian trait, governed by one genetic variant, on the basis of 1000 simulated Mendelian traits for each dataset. Overall, the unique causal genetic variant was identified as significant for all 1000 runs, with the exception of seven runs performed with the sake subpopulation, which clearly illustrates the high capacity of identifying the causal SNP for all datasets in case of a Mendelian trait. Equally important is the ability of the dataset to differentiate the causal variant from other markers. For the evaluation of this parameter, we computed the genomic inflation factor (*λ*), which quantifies the extent of the bulk inflation and the excess of false positive cases. All datasets except the sake one have *λ* close to the expected value of 1 for most runs, indicating that they are well suited for detecting and mapping of Mendelian traits (electronic supplementary material, figure S1). The sake dataset shows much more scattered *λ* values, revealing a certain lack of power to efficiently detect associations.

**Figure 2.**
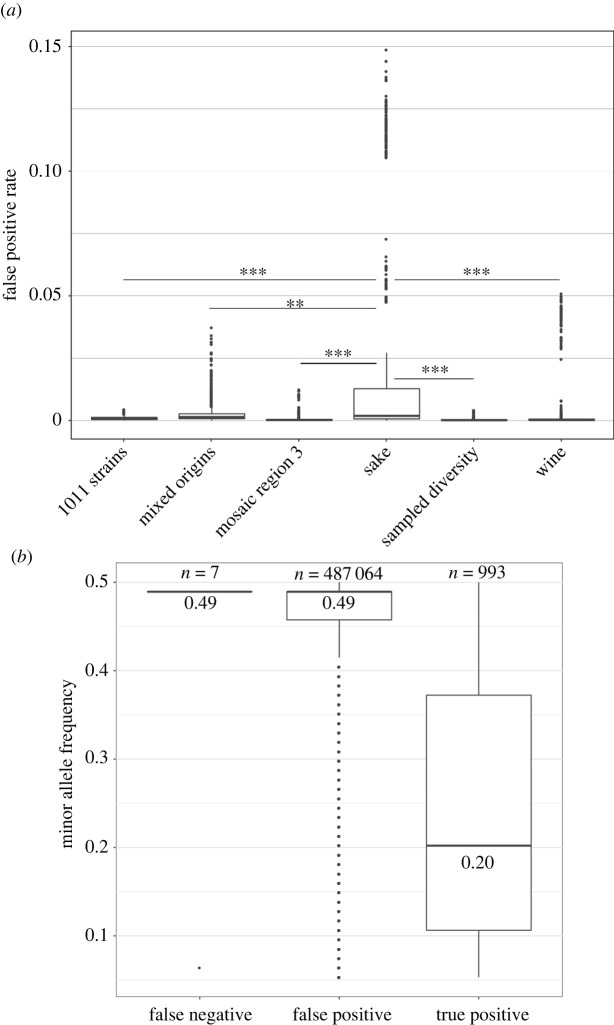
Mapping of Mendelian traits. (*a*) Distribution of the false positive rate observed across the subpopulations for the GWAS performed on 1000 simulated Mendelian traits. A Kruskal-Wallis test indicates that the samples do not share the same distribution. We also tested whether the sake distribution was significantly different compared to all the other datasets using a Mann-Whitney-Wilcoxon test (**p-value < 2 × 10^−8^; ***p-value < 2 × 10^−16^). (*b*) Minor allele frequency distribution of the false negative, false positive and true positive variants detected by GWAS with the sake subpopulation, for which a bias toward variants with MAF = 0.49 is observed.

This observation leads us to determine the proportion of false positives among the variants detected as associated. The median value of the false positive rate (FPR) is very low for all datasets (<2 × 10^−3^), but can vary a lot among a run to another (figure 2*a*; electronic supplementary material, table S2). The higher values are observed for the sake dataset, for which 145 runs show a FPR of more than 10%, making the results from this dataset unreliable. To a lesser extent, some runs of the European wine dataset reach a value of 5%, which is also much higher than expected for a high-confidence dataset. However, these high FPRs can be considered overestimated when there is linkage between some markers and the causal genetic variant (electronic supplementary material, figure S2).

Although the causal genetic variant governing a Mendelian trait is always found with the exception of a few runs, false positives rates can vary between the datasets that were tested. These results suggest that our datasets appear globally suitable for performing GWAS in the case of a low genetic complexity trait. Indeed, we do not notice any inflated *p*-values due to the population structure, except for the sake subpopulation, showing as a result a very large number of false positives. The sample size is not the only factor influencing the FPR, as the sampled diversity shows the best performance, and the European wine cluster (second highest sample size) performs poorly, which means that a careful selection of strains to avoid the overrepresentation of some closely related strains could be a good way to create an effective dataset for GWAS.

### (c) Relatedness and the mapping of Mendelian traits: the example of the sake subpopulation

The population corresponding to the cluster of sake strains shows relatively poor performance, even for a Mendelian trait. Indeed, it corresponds to the only dataset for which the causal genetic variant has not been associated for some runs and, more importantly, it shows high FPR for many runs preventing any conclusion to be drawn from association studies. Several characteristics of this dataset can be attributed to this failure. First, this dataset only contains 47 strains, but sample size cannot be the only factor, as the performance of the dataset does not seem to be related to the size for the other datasets. We also observed that the global genetic diversity within this cluster in very low (π = 0.0008). Moreover, 23 out of the 47 strains are clustered on a thin branch of the tree (figure 1*a*), suggesting that they share a very recent common ancestor that could be the source of a systematic bias in allele frequency such as the overrepresentation of variants with a MAF around 0.49 (figure 1*b*). Across the 1000 associations, we observed that these variants were overrepresented in both the false negative and false positive categories (figure 2*b*). Among the 135 simulated traits for which the causal SNP had a MAF around 0.49, our test failed to associate six and a higher number of FP was observed compared to the others (electronic supplementary material, figure S3).

In general, it seems that the genetic variants with a MAF around 0.49 will cause problems. If they are causal, it will be more difficult to detect them by association or will lead to an increase in the number of false positives. If they are not causal, they will still be wrongly associated with the trait. This case allowed us to measure the impact of relatedness (i.e. sharing a recent common ancestor) on the mapping of traits with GWAS, as the relationship will result in a MAF bias of the genetic variants.

### (d) Mapping of complex traits and the importance of sample size

To further assess the performance of our datasets, we tested their utility to map causal SNPs in the context of 1000 simulated complex traits governed by 10 SNPs for each dataset and estimated the fraction of heritability recovered. No dataset could detect all the causal SNPs that were used to simulate the phenotypes. The best results obtained are the detection of eight causal SNPs for 15 runs of the 1011 strains dataset. The median of the true positive rate (TPR) for this dataset is also the best, with five true positive SNPs out of 10 detected (TPR = 0.5). It seems that the TPR increases with the size of the sample, indicating that this parameter probably plays an important role in the detection power for a complex trait (electronic supplementary material, figure S4). To further test this, we downsampled the mixed, sampled diversity and wine datasets to match the number of individuals composing the sake population. The genetic diversity of these new populations matched the original populations, allowing us to assess only the effect of reduced population size. The GWAS simulations performed very poorly, with a median of the TPR of 0 for all three subpopulations and a maximum of 0.2 for 10 out of 1000 runs for the subsampled wine population (electronic supplementary material, figure S5). These results suggest that sample size correlates with performance of association studies, although it is not the only factor influencing the outcome (electronic supplementary material, figure S5).

**Figure 3.**
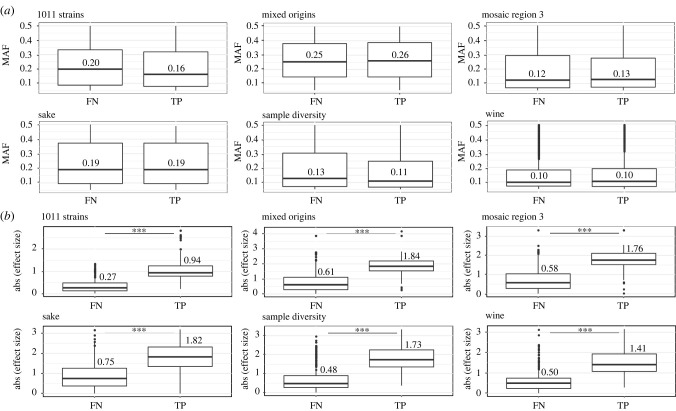
Mapping of complex traits. (*a*) Distribution of the minor allele frequency (MAF) of the false negative (FN) and true positive (TP) variants detected by GWAS among the 1000 simulated complex traits across the subpopulations. (*b*) Distribution of the absolute effect size of the FN and TP variants detected by GWAS among the 1000 simulated complex traits across the subpopulations.

The MAF of causal SNPs does not influence their detection propensity (figure 3*a*). Rather, detection propensity increased with the effect size (figure 3*b*). This suggests that, as the genetic contribution to the phenotype is distributed over several SNPs, the smaller the effect size of a genetic variant, the more difficult it will be to detect. In addition, the *p*-values of the causal genetic variants are positively correlated with the effect sizes for all datasets, indicating that variants with high effect sizes are more likely to have high association scores (electronic supplementary material, figure S6). Taken together, these results illustrate how the missing heritability can be hidden behind a large number of variants with small effects and that datasets with a larger sample size are more likely to detect the causal variants.

### (e) Dataset composition impacts the false positive rate

Interestingly, the median FPR in the case of complex traits is lower than when we simulated a Mendelian trait (electronic supplementary material, figure S7, table S3) and is less than 4 × 10^−4^ for all datasets. Based on the FPR distribution, the sampled diversity and the 1011 strains datasets are the least likely to detect spurious associations. The sake dataset has the most outliers, with 17 of them being above 10%. This proportion is surprisingly better than for the mapping of a Mendelian trait, but still very high. The distribution of the genomic inflation factor *λ* of the 1000 runs per dataset (electronic supplementary material, figure S8) indicates that most datasets have *λ* values closely centred on 1. The sake dataset appears once again as an outlier with a much broadly distributed value of *λ*, confirming that a high proportion of false positives can be expected with this dataset.

We observed a tendency for FPR to increase as the TPR increases, illustrating the intuitive fact that causal variants can be mapped by chance if there are many SNPs that have been significantly identified. However, this tendency is less marked as the sample size increases (electronic supplementary material, figure S9), which is consistent with the previous result showing that a large sample size will reduce the false positive rate.

These results show that the sample size clearly influences the GWAS result, in particular in the case of a complex trait but also that this parameter is not the only one to influence the GWAS result as the sampled diversity shows very good results with only 133 individuals.

### (f) Association mapping using real data

To test associations with actual phenotypic values, we measured growth for all our isolates on 10 mM copper sulfate and normalized these sizes by growth on the standard YPD medium at 30°C. These measures are a proxy for the fitness of each isolate on stress induced by copper sulfate. This phenotype shows a high genome-wide heritability for the full dataset (83.78%), which is of the same order of magnitude as the heritability used to simulate the phenotypes in this study. The gene *CUP1-2* is the main copper-activated metallothionine in *S. cerevisiae*. The protein Cup1p binds and sequesters copper(I), Cu+, allowing the cell to control copper ion homeostasis. This ion, although essential for the survival of yeasts, is also an environmental heavy metal toxicant at high concentrations, which is used, for example, to kill downy mildew in vineyards, where *S. cerevisiae* is often found. Tandem duplications of the *CUP1* gene are frequent in budding yeasts, and the tolerance to copper ion is correlated with the number of copies of this gene [17]. As the copy number variation of the *CUP1-2* gene is known to be of major importance for this trait, it is expected that association with well-suited datasets should easily detect this genetic variant.

The association was performed with the same method as that used for the simulations, with a dataset-specific threshold determined by 100 runs of permutations. The copy number variant of *CUP1-2* was not detected in the sake, the mixed origins and the mosaic regions 3 datasets (electronic supplementary material, table S4). The mosaic region 3 and the sake datasets also showed associations with other variants that are not known to be involved in the resistance to copper sulfate. While the involvement of these genes in this phenotype cannot be ruled out, one should also keep in mind that these datasets had high FPR for some simulations, especially the sake dataset. In the other datasets, the *CUP1-2* gene was significantly associated with the phenotype, with no other significant association.

The score of association is an indicator of the ease with which a dataset is able to detect the causal variant. Here, we see that the 1011 strains dataset shows the lowest *p*-value (4.9 × 10^−44^), followed by the European wine dataset (6.03 × 10^−18^) and the sampled diversity dataset (1.04 × 10^−14^). These results are consistent with our simulations, as the 1011 strains and the sampled diversity datasets were the ones that showed the best results. The detection of the *CUP1-2* gene for the European wine subpopulation is attributed to the evolutionary history of this population, and its link with human activity. Indeed, in that case, the acquisition of resistance to copper sulfate is certainly reflecting convergent evolution due to human selection for industrial processes [28], thus emphasizing the fact that dataset composition also depends on the phenotype of interest.

## 4. Discussion

In this work, we performed extensive GWAS simulations by measuring the ability of linear mixed models to find a significant association between causal genetic variants and simulated phenotypes of each individual. These tests have been performed on five subpopulations as well as the complete dataset of the 1011 *S. cerevisiae* isolates [4]. While variants are easily mapped for Mendelian traits regardless the used dataset, variance in terms of type-1 error can be observed. This variability might not only be related to sample size, but to other variables such as close relatedness between isolates. However, further simulations are needed to explore this aspect. Regarding the mapping of complex traits, a variation between datasets is observed in terms of detection power. Indeed, while the 1011 strains dataset shows the best performances, the sampled diversity dataset shows a low type-1 error rate, even though its sample size is small compared to the European wine cluster and the 1011 strains dataset, indicating that confounding factors can also be handled with well-suited datasets. Finally, we tried association mapping using a real phenotype, more specifically on the ability to grow in the presence of copper sulfate, and checked if we could identify the already known *CUP1-2* gene responsible for the variation in fitness. This genetic variant was successfully identified in three datasets without any other association while it was missed in the other three. The detection of the causal variant was consistent with our simulations and showed us that specific populations could represent good datasets to perform associations with given phenotypes.

GWAS studies have been problematic in *S. cerevisiae* due to the highly stratified populations, which led to high type-1 error rates [14,15]. Here, we showed that structure is well taken into account in most of the datasets by a LMM approach because the type-1 error rates are low for most of them. Our hypothesis is that sample size was the limiting factor in the aforementioned studies. Indeed, the type-1 error rates of our datasets with the smallest sample sizes were among the highest. But our results support the fact that other parameters have an impact on the GWAS outcomes, as the performance does not always correlate with the sample size. For example, it seems that it is important to build datasets with individuals that do not share a recent common ancestor, as this kinship will introduce a bias in allele frequencies which will also lead to false associations, as we have observed for the sake and the European wine subpopulations. In fact, additional simulations would be very useful in order to test more precisely for other variables that could have an impact on the results of the GWAS, such as genetic diversity, variation in heritability and size effect distribution.

The results of our associations vary considerably from one cohort to another, allowing us to evaluate the limits of GWAS and present what would constitute an ideal dataset for association studies. For mapping a Mendelian trait, the sampled diversity set is the best, with the fewest false positives, and is followed by the total dataset with 1011 strains. When the trait is complex, the entire population has the advantage of mapping more causal variants than any other. In addition, the false positive rate is close to that we observe for the sampled diversity dataset, which has, as for a Mendelian trait, the lowest value. It is now evident that the sake cluster is poorly suited to perform GWAS. First, the number of sequences composing this dataset is too low and therefore does not provide sufficient statistical power for trustworthy associations. Second, the strains composing this cluster are very close to each other and all share large parts of the genome due to the fact that the common ancestor is very recent compared to other datasets. We initially thought that the European wine subpopulation, being a clear lineage and composed of a high number of strains (*n* = 323), would constitute a good set to perform GWAS because the population is not stratified. The number of false positives by run is very high for a Mendelian and complex trait and the results obtained with this dataset should therefore be taken with caution.

To go further, it would be interesting to test whether larger sample sizes would improve the detection of causal loci. Indeed, since there is a skew toward low-frequency alleles in the yeast population, a dataset with a larger sample size might be able to identify rarer genetic variants, and therefore increase the proportion of phenotypic variation explained. Increasing the sample size will also allow more subsets to be created, while maintaining a high sample size, and could be of great help when association is tested with a specific phenotype. Another solution is to perform GWAS on an inbred population resulting from a diallel cross [29], i.e. all the pairwise crosses between parental accessions for which the genomic sequence is known. This strategy offers several advantages that may be worth to consider for further study.

## Data Availability

The GWAS matrices in gvcf format as well as scripts used in this study are provided here: http://1002genomes.u-strasbg.fr/files/GWAS_simulations/.
